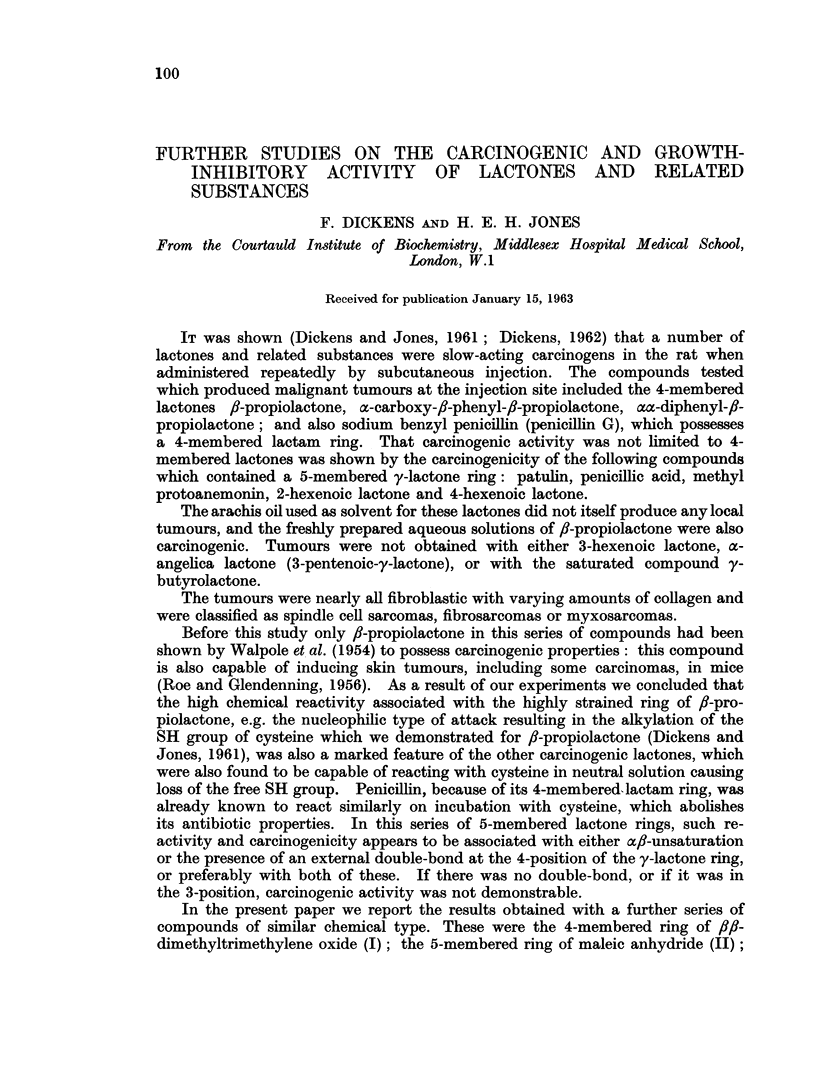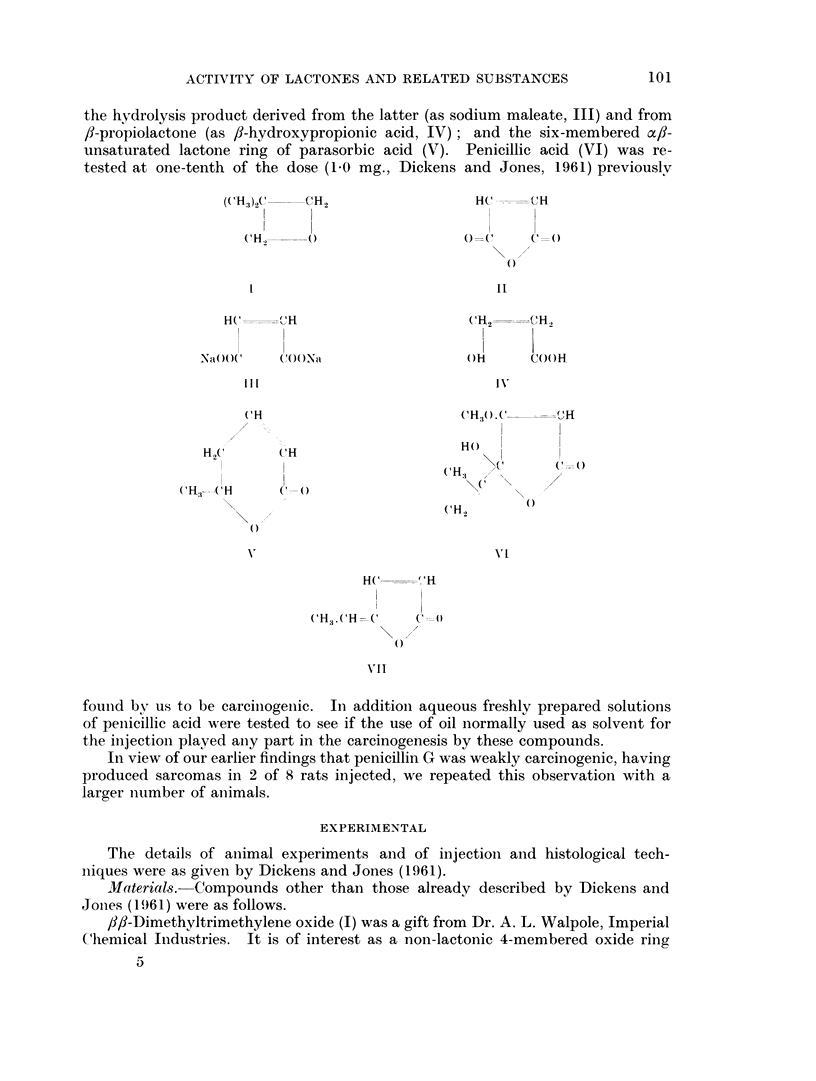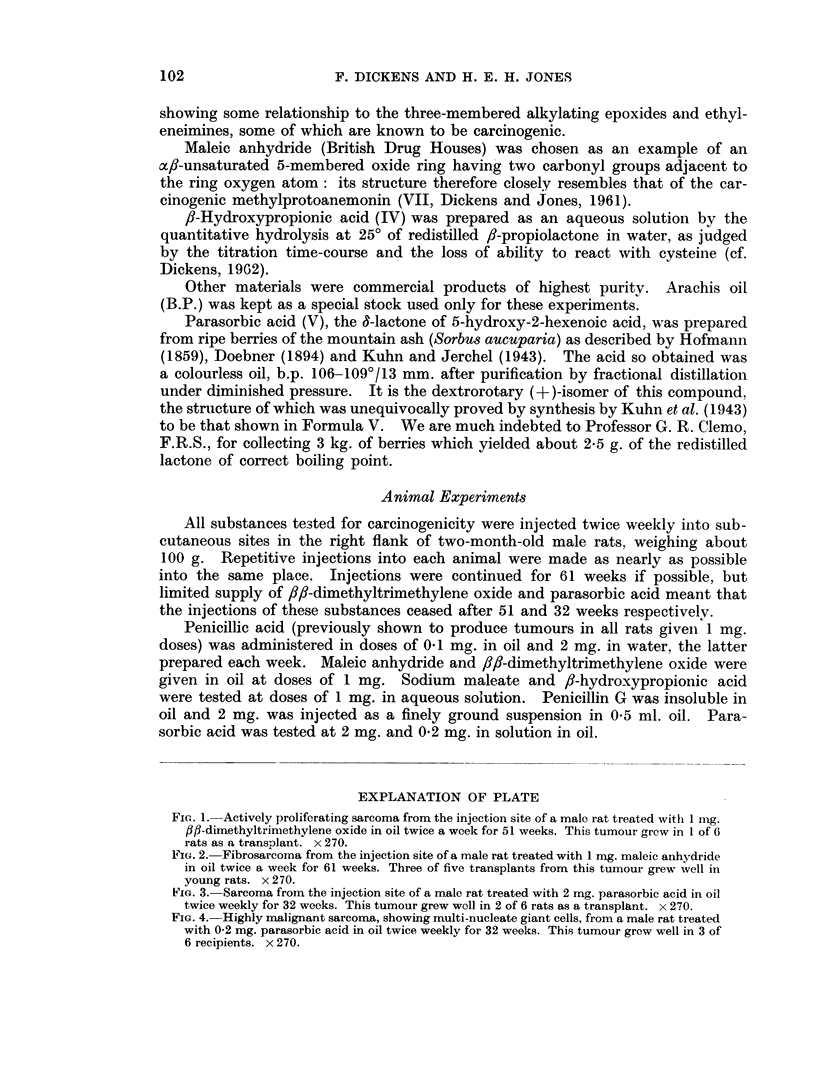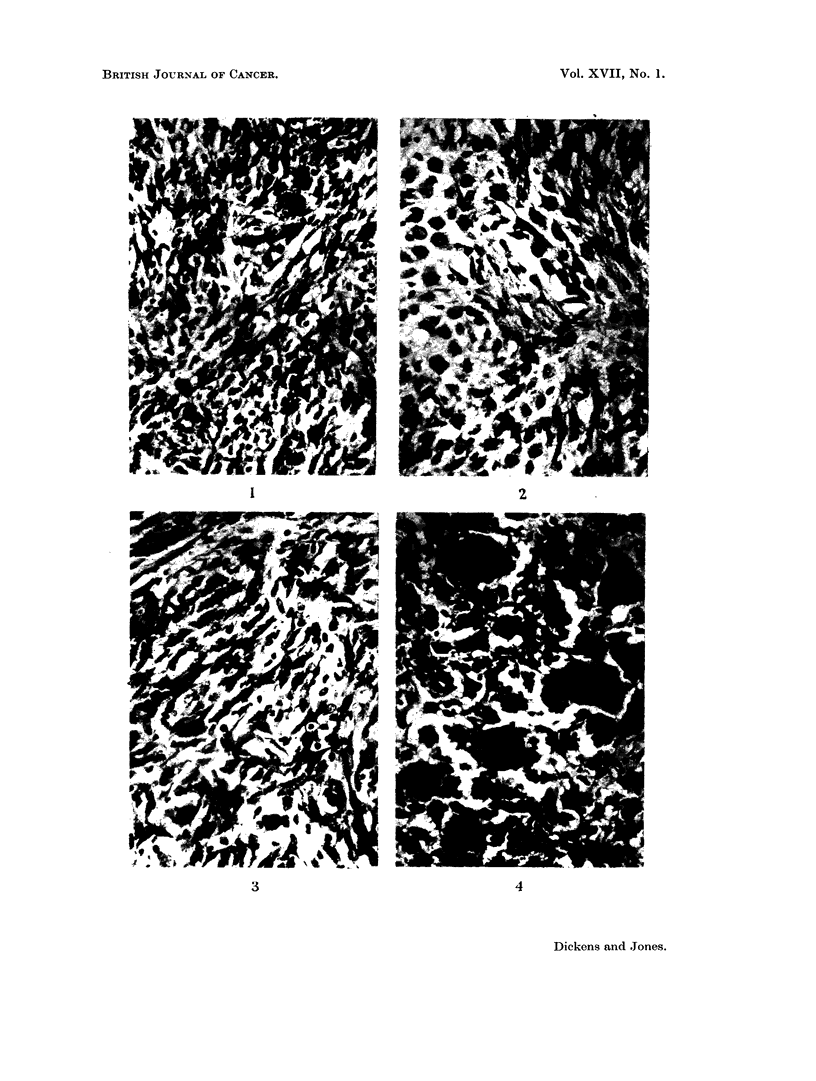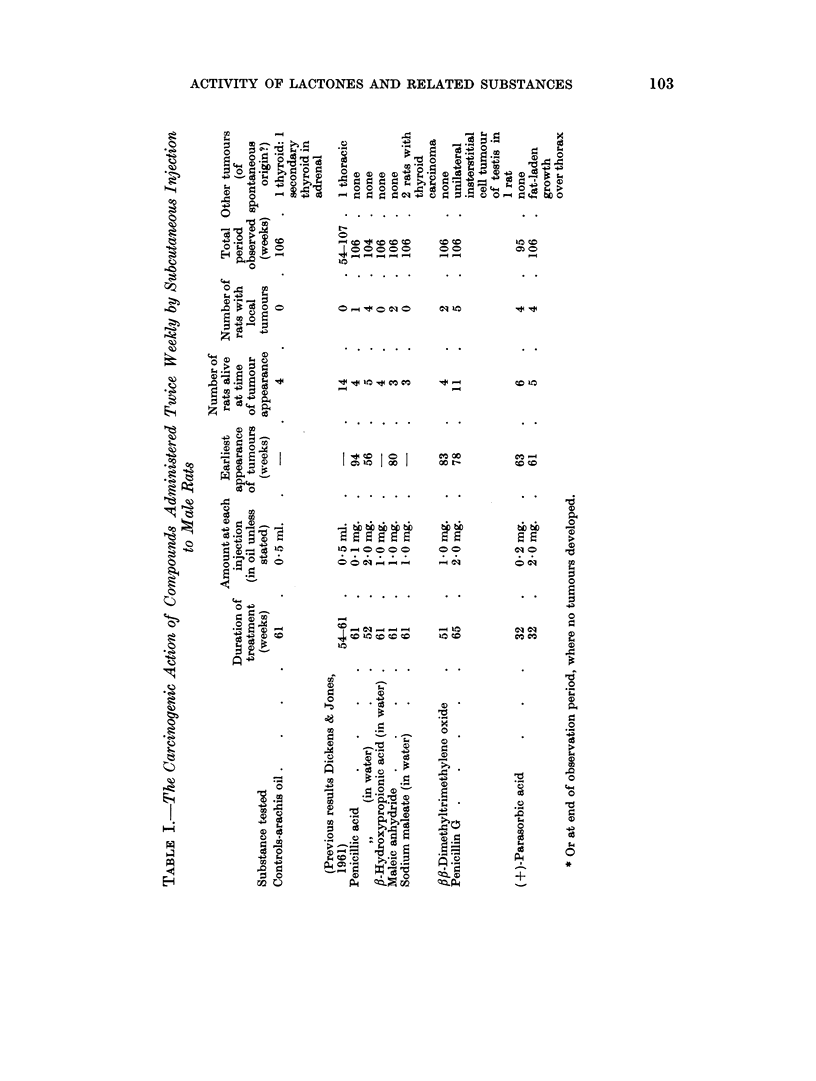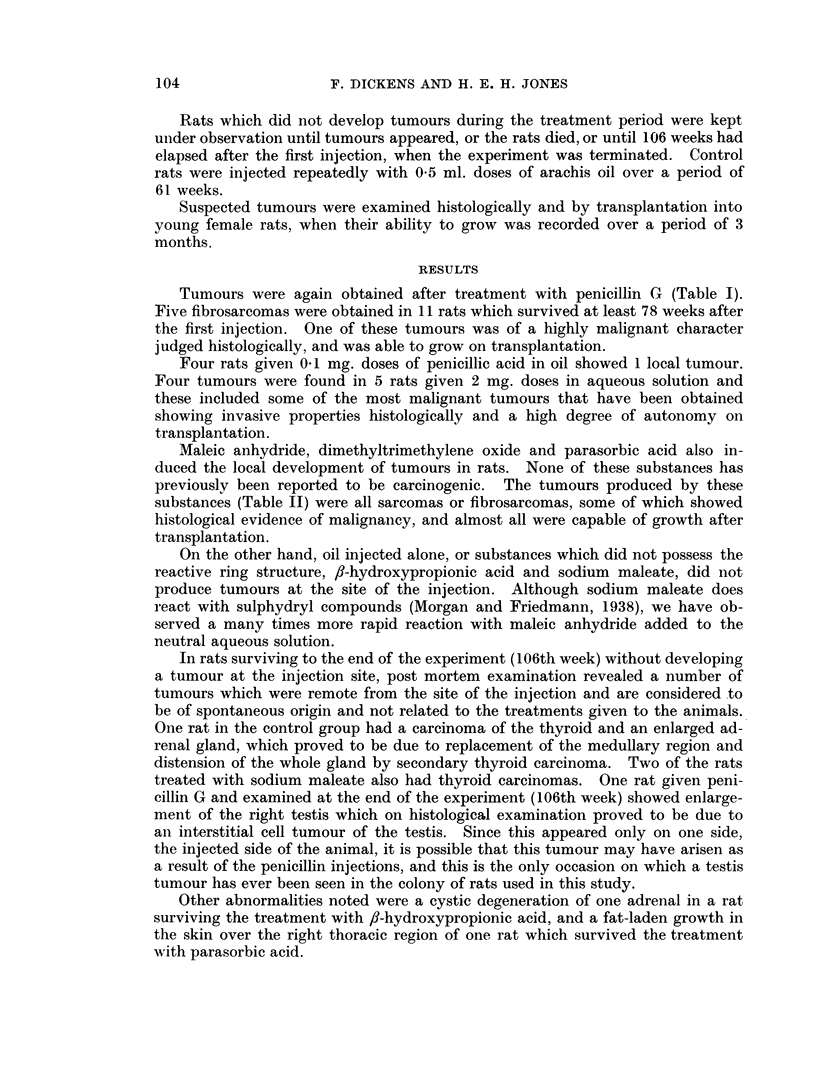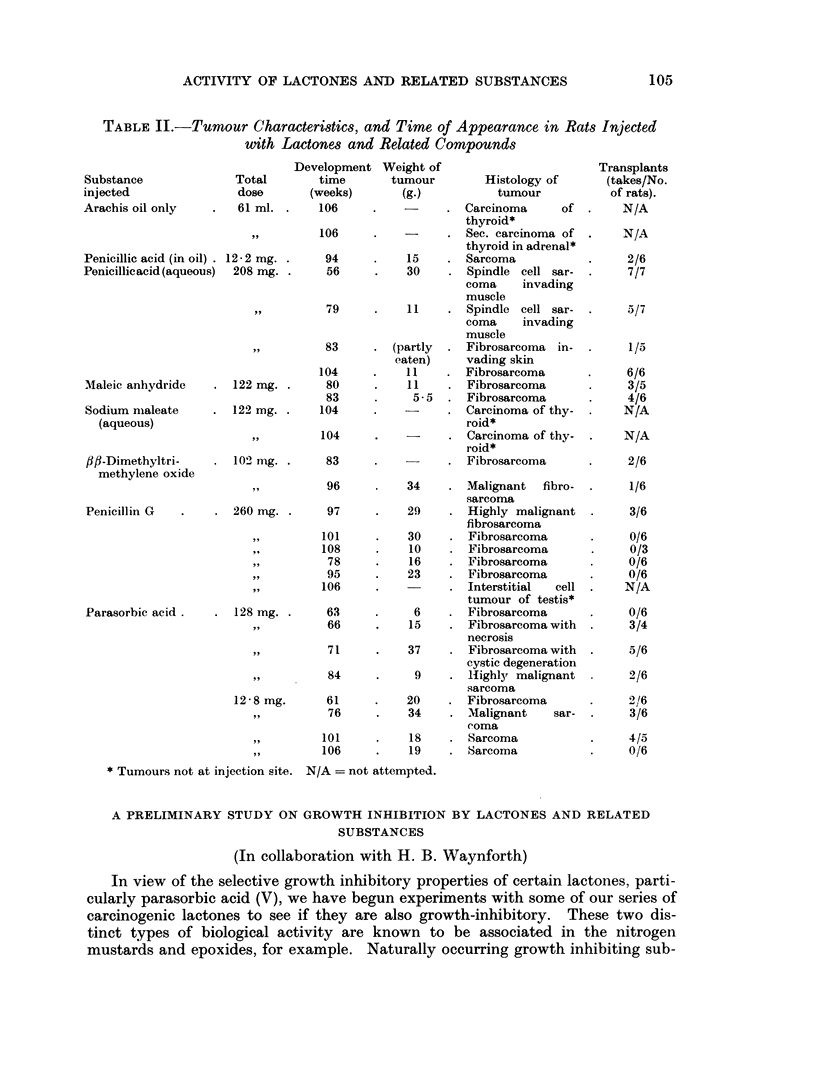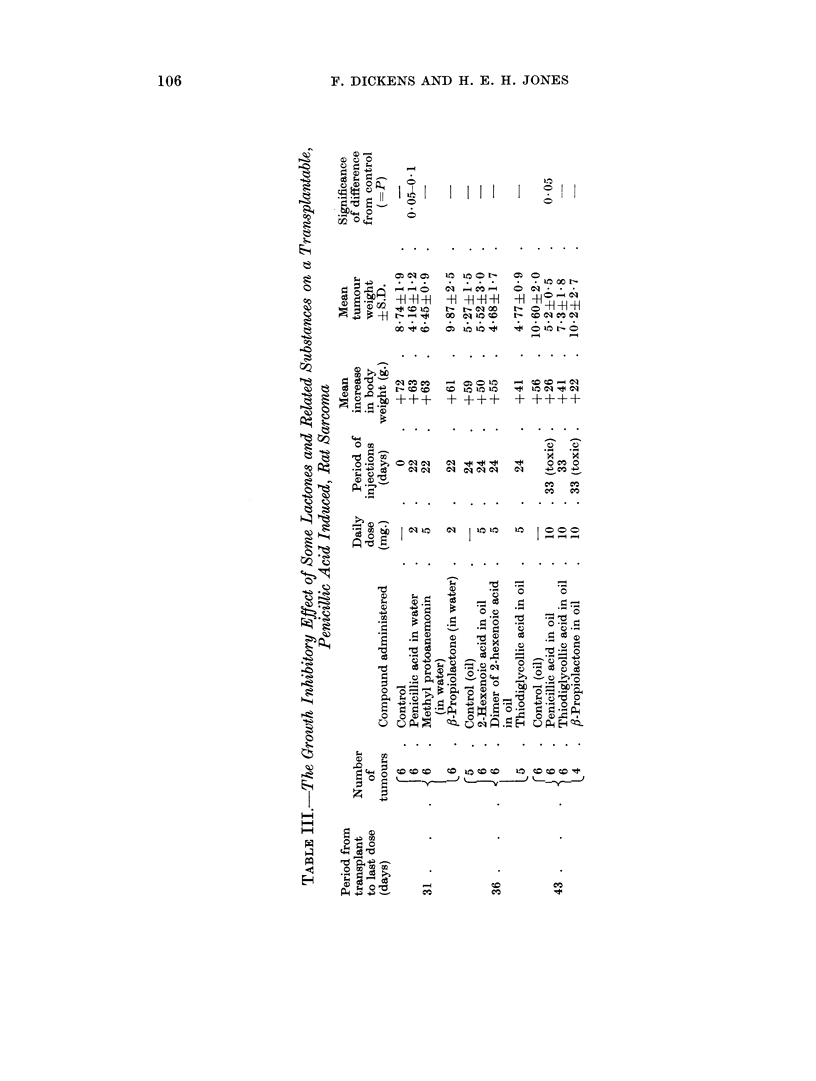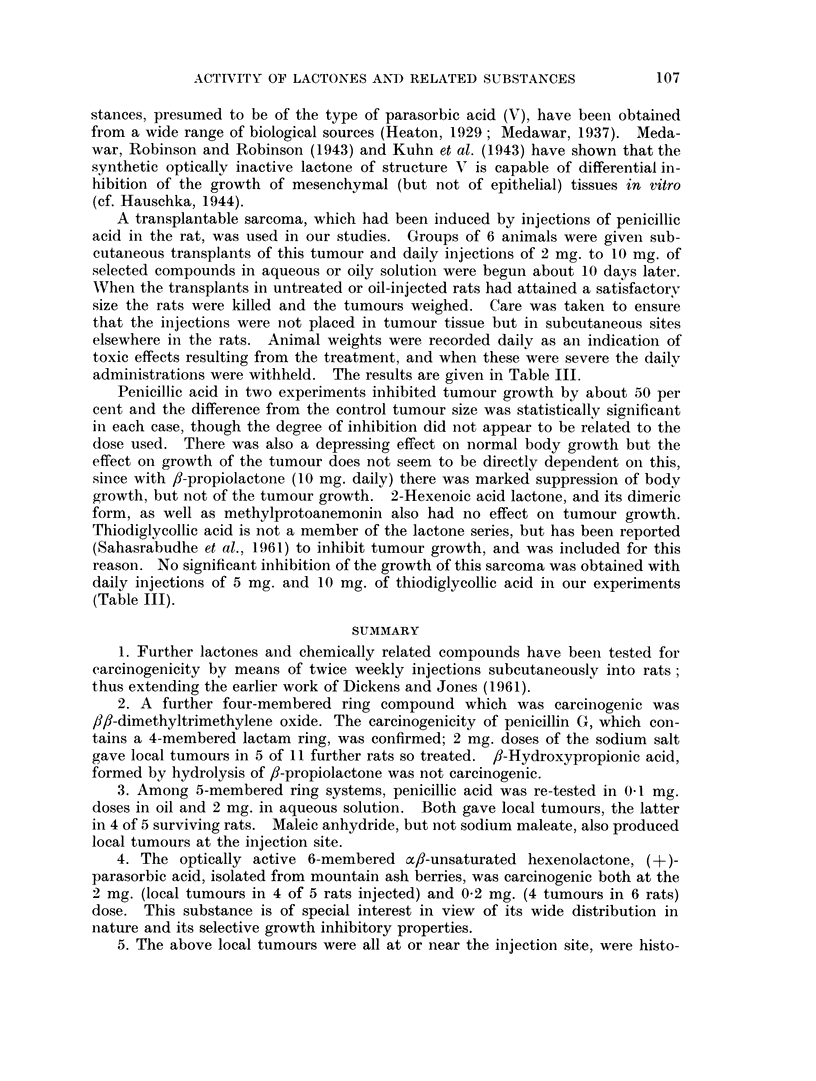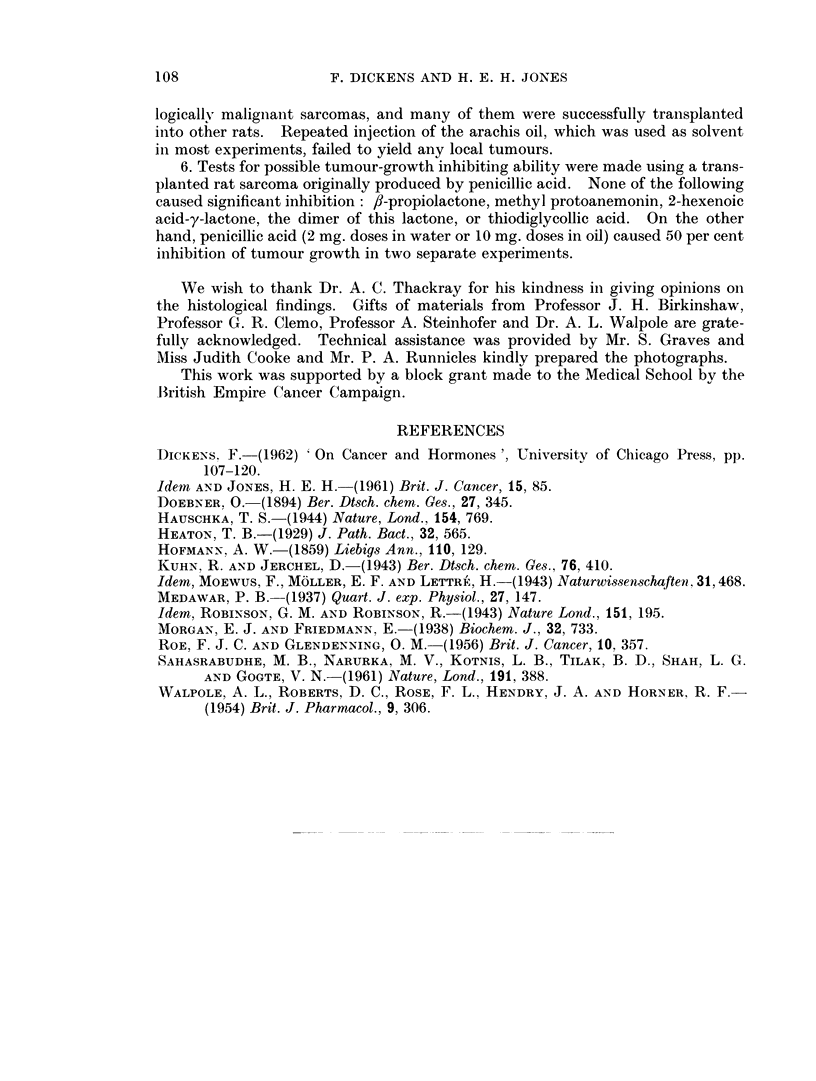# Further Studies on the Carcinogenic and Growth-Inhibitory Activity of Lactones and Related Substances

**DOI:** 10.1038/bjc.1963.15

**Published:** 1963-03

**Authors:** F. Dickens, H. E. H. Jones

## Abstract

**Images:**


### FURTHER STUDIES ON THE CARCINOGENIC AND GROWTH-INHIBITORY ACTIVITY OF LACTONES AND RELATED SUBSTANCES

F. DICKENS AND H. E. H. JONES

*From the Courtauld Institute of Biochemistry, Middlesex Hospital Medical School, London, W.1*

Received for publication January 15, 1963

IT was shown (Dickens and Jones, 1961 ; Dickens, 1962) that a number of lactones and related substances were slow-acting carcinogens in the rat when administered repeatedly by subcutaneous injection. The compounds tested which produced malignant tumours at the injection site included the 4-membered lactones $\beta$-propiolactone, $\alpha$-carboxy-$\beta$-phenyl-$\beta$-propiolactone, $\alpha\alpha$-diphenyl-$\beta$-propiolactone ; and also sodium benzyl penicillin (penicillin G), which possesses a 4-membered lactam ring. That carcinogenic activity was not limited to 4-membered lactones was shown by the carcinogenicity of the following compounds which contained a 5-membered $\gamma$-lactone ring : patulin, penicillic acid, methyl protoanemonin, 2-hexenoic lactone and 4-hexenoic lactone.

The arachis oil used as solvent for these lactones did not itself produce any local tumours, and the freshly prepared aqueous solutions of $\beta$-propiolactone were also carcinogenic. Tumours were not obtained with either 3-hexenoic lactone, $\alpha$-angelica lactone (3-pentenoic-$\gamma$-lactone), or with the saturated compound $\gamma$-butyrolactone.

The tumours were nearly all fibroblastic with varying amounts of collagen and were classified as spindle cell sarcomas, fibrosarcomas or myxosarcomas.

Before this study only $\beta$-propiolactone in this series of compounds had been shown by Walpole *et al.* (1954) to possess carcinogenic properties : this compound is also capable of inducing skin tumours, including some carcinomas, in mice (Roe and Glendenning, 1956). As a result of our experiments we concluded that the high chemical reactivity associated with the highly strained ring of $\beta$-propiolactone, e.g. the nucleophilic type of attack resulting in the alkylation of the SH group of cysteine which we demonstrated for $\beta$-propiolactone (Dickens and Jones, 1961), was also a marked feature of the other carcinogenic lactones, which were also found to be capable of reacting with cysteine in neutral solution causing loss of the free SH group. Penicillin, because of its 4-membered lactam ring, was already known to react similarly on incubation with cysteine, which abolishes its antibiotic properties. In this series of 5-membered lactone rings, such reactivity and carcinogenicity appears to be associated with either $\alpha\beta$-unsaturation or the presence of an external double-bond at the 4-position of the $\gamma$-lactone ring, or preferably with both of these. If there was no double-bond, or if it was in the 3-position, carcinogenic activity was not demonstrable.

In the present paper we report the results obtained with a further series of compounds of similar chemical type. These were the 4-membered ring of $\beta\beta$-dimethyltrimethylene oxide (I) ; the 5-membered ring of maleic anhydride (II) ; the hydrolysis product derived from the latter (as sodium maleate, III) and from $\beta$-propiolactone (as $\beta$-hydroxypropionic acid, IV); and the six-membered $\alpha\beta$-unsaturated lactone ring of parasorbic acid (V). Penicillic acid (VI) was re-tested at one-tenth of the dose (1·0 mg., Dickens and Jones, 1961) previously

I

II

III

IV

V

VI

VII

found by us to be carcinogenic. In addition aqueous freshly prepared solutions of penicillic acid were tested to see if the use of oil normally used as solvent for the injection played any part in the carcinogenesis by these compounds.

In view of our earlier findings that penicillin G was weakly carcinogenic, having produced sarcomas in 2 of 8 rats injected, we repeated this observation with a larger number of animals.

### EXPERIMENTAL

The details of animal experiments and of injection and histological techniques were as given by Dickens and Jones (1961).

*Materials.*—Compounds other than those already described by Dickens and Jones (1961) were as follows.

$\beta\beta$-Dimethyltrimethylene oxide (I) was a gift from Dr. A. L. Walpole, Imperial Chemical Industries. It is of interest as a non-lactonic 4-membered oxide ring showing some relationship to the three-membered alkylating epoxides and ethyleneimines, some of which are known to be carcinogenic.

Maleic anhydride (British Drug Houses) was chosen as an example of an $\alpha\beta$-unsaturated 5-membered oxide ring having two carbonyl groups adjacent to the ring oxygen atom : its structure therefore closely resembles that of the carcinogenic methylprotoanemonin (VII, Dickens and Jones, 1961).

$\beta$-Hydroxypropionic acid (IV) was prepared as an aqueous solution by the quantitative hydrolysis at 25° of redistilled $\beta$-propiolactone in water, as judged by the titration time-course and the loss of ability to react with cysteine (cf. Dickens, 1962).

Other materials were commercial products of highest purity. Arachis oil (B.P.) was kept as a special stock used only for these experiments.

Parasorbic acid (V), the $\delta$-lactone of 5-hydroxy-2-hexenoic acid, was prepared from ripe berries of the mountain ash (*Sorbus aucuparia*) as described by Hofmann (1859), Doebner (1894) and Kuhn and Jerchel (1943). The acid so obtained was a colourless oil, b.p. 106–109°/13 mm. after purification by fractional distillation under diminished pressure. It is the dextrorotary (+)-isomer of this compound, the structure of which was unequivocally proved by synthesis by Kuhn *et al.* (1943) to be that shown in Formula V. We are much indebted to Professor G. R. Clemo, F.R.S., for collecting 3 kg. of berries which yielded about 2·5 g. of the redistilled lactone of correct boiling point.

### *Animal Experiments*

All substances tested for carcinogenicity were injected twice weekly into subcutaneous sites in the right flank of two-month-old male rats, weighing about 100 g. Repetitive injections into each animal were made as nearly as possible into the same place. Injections were continued for 61 weeks if possible, but limited supply of $\beta\beta$-dimethyltrimethylene oxide and parasorbic acid meant that the injections of these substances ceased after 51 and 32 weeks respectively.

Penicillic acid (previously shown to produce tumours in all rats given 1 mg. doses) was administered in doses of 0·1 mg. in oil and 2 mg. in water, the latter prepared each week. Maleic anhydride and $\beta\beta$-dimethyltrimethylene oxide were given in oil at doses of 1 mg. Sodium maleate and $\beta$-hydroxypropionic acid were tested at doses of 1 mg. in aqueous solution. Penicillin G was insoluble in oil and 2 mg. was injected as a finely ground suspension in 0·5 ml. oil. Parasorbic acid was tested at 2 mg. and 0·2 mg. in solution in oil.

---

EXPLANATION OF PLATE

FIG. 1.—Actively proliferating sarcoma from the injection site of a male rat treated with 1 mg. $\beta\beta$-dimethyltrimethylene oxide in oil twice a week for 51 weeks. This tumour grew in 1 of 6 rats as a transplant. $\times$270.

FIG. 2.—Fibrosarcoma from the injection site of a male rat treated with 1 mg. maleic anhydride in oil twice a week for 61 weeks. Three of five transplants from this tumour grew well in young rats. $\times$270.

FIG. 3.—Sarcoma from the injection site of a male rat treated with 2 mg. parasorbic acid in oil twice weekly for 32 weeks. This tumour grew well in 2 of 6 rats as a transplant. $\times$270.

FIG. 4.—Highly malignant sarcoma, showing multi-nucleate giant cells, from a male rat treated with 0·2 mg. parasorbic acid in oil twice weekly for 32 weeks. This tumour grew well in 3 of 6 recipients. $\times$270.

1

2

3

4

Dickens and Jones.

TABLE I.—*The Carcinogenic Action of Compounds Administered Twice Weekly by Subcutaneous Injection to Male Rats*

| Substance tested | Duration of treatment (weeks) | Amount at each injection (in oil unless stated) | Earliest appearance of tumours (weeks) | Number of rats alive at time of tumour appearance | Number of rats with local tumours | Total period observed (weeks) | Other tumours (of spontaneous origin?) |
|---|---|---|---|---|---|---|---|
| Controls-arachis oil | 61 | 0·5 ml. | — | 4 | 0 | 106 | 1 thyroid: 1 secondary thyroid in adrenal |
| (Previous results Dickens & Jones, 1961) | 54–61 | 0·5 ml. | — | 14 | 0 | 54–107 | 1 thoracic |
| Penicillic acid | 61 | 0·1 mg. | 94 | 4 | 1 | 106 | none |
| ,, (in water) | 52 | 2·0 mg. | 56 | 5 | 4 | 104 | none |
| β-Hydroxypropionic acid (in water) | 61 | 1·0 mg. | — | 4 | 0 | 106 | none |
| Maleic anhydride | 61 | 1·0 mg. | 80 | 3 | 2 | 106 | none |
| Sodium maleate (in water) | 61 | 1·0 mg. | — | 3 | 0 | 106 | 2 rats with thyroid carcinoma |
| ββ-Dimethyltrimethylene oxide | 51 | 1·0 mg. | 83 | 4 | 2 | 106 | none |
| Penicillin G | 65 | 2·0 mg. | 78 | 11 | 5 | 106 | unilateral insterstitial cell tumour of testis in 1 rat |
| (+)-Parasorbic acid | 32 | 0·2 mg. | 63 | 6 | 4 | 95 | none |
| | 32 | 2·0 mg. | 61 | 5 | 4 | 106 | fat-laden growth over thorax |

* Or at end of observation period, where no tumours developed.

Rats which did not develop tumours during the treatment period were kept under observation until tumours appeared, or the rats died, or until 106 weeks had elapsed after the first injection, when the experiment was terminated. Control rats were injected repeatedly with 0·5 ml. doses of arachis oil over a period of 61 weeks.

Suspected tumours were examined histologically and by transplantation into young female rats, when their ability to grow was recorded over a period of 3 months.

### RESULTS

Tumours were again obtained after treatment with penicillin G (Table I). Five fibrosarcomas were obtained in 11 rats which survived at least 78 weeks after the first injection. One of these tumours was of a highly malignant character judged histologically, and was able to grow on transplantation.

Four rats given 0·1 mg. doses of penicillic acid in oil showed 1 local tumour. Four tumours were found in 5 rats given 2 mg. doses in aqueous solution and these included some of the most malignant tumours that have been obtained showing invasive properties histologically and a high degree of autonomy on transplantation.

Maleic anhydride, dimethyltrimethylene oxide and parasorbic acid also induced the local development of tumours in rats. None of these substances has previously been reported to be carcinogenic. The tumours produced by these substances (Table II) were all sarcomas or fibrosarcomas, some of which showed histological evidence of malignancy, and almost all were capable of growth after transplantation.

On the other hand, oil injected alone, or substances which did not possess the reactive ring structure, $\beta$-hydroxypropionic acid and sodium maleate, did not produce tumours at the site of the injection. Although sodium maleate does react with sulphydryl compounds (Morgan and Friedmann, 1938), we have observed a many times more rapid reaction with maleic anhydride added to the neutral aqueous solution.

In rats surviving to the end of the experiment (106th week) without developing a tumour at the injection site, post mortem examination revealed a number of tumours which were remote from the site of the injection and are considered to be of spontaneous origin and not related to the treatments given to the animals. One rat in the control group had a carcinoma of the thyroid and an enlarged adrenal gland, which proved to be due to replacement of the medullary region and distension of the whole gland by secondary thyroid carcinoma. Two of the rats treated with sodium maleate also had thyroid carcinomas. One rat given penicillin G and examined at the end of the experiment (106th week) showed enlargement of the right testis which on histological examination proved to be due to an interstitial cell tumour of the testis. Since this appeared only on one side, the injected side of the animal, it is possible that this tumour may have arisen as a result of the penicillin injections, and this is the only occasion on which a testis tumour has ever been seen in the colony of rats used in this study.

Other abnormalities noted were a cystic degeneration of one adrenal in a rat surviving the treatment with $\beta$-hydroxypropionic acid, and a fat-laden growth in the skin over the right thoracic region of one rat which survived the treatment with parasorbic acid.

TABLE II.—*Tumour Characteristics, and Time of Appearance in Rats Injected with Lactones and Related Compounds*

| Substance injected | Total dose | Development time (weeks) | Weight of tumour (g.) | Histology of tumour | Transplants (takes/No. of rats). |
|---|---|---|---|---|---|
| Arachis oil only | 61 ml. | 106 | — | Carcinoma of thyroid* | N/A |
| | ,, | 106 | — | Sec. carcinoma of thyroid in adrenal* | N/A |
| Penicillic acid (in oil) | 12·2 mg. | 94 | 15 | Sarcoma | 2/6 |
| Penicillic acid (aqueous) | 208 mg. | 56 | 30 | Spindle cell sarcoma invading muscle | 7/7 |
| | ,, | 79 | 11 | Spindle cell sarcoma invading muscle | 5/7 |
| | ,, | 83 | (partly eaten) | Fibrosarcoma invading skin | 1/5 |
| | | 104 | 11 | Fibrosarcoma | 6/6 |
| Maleic anhydride | 122 mg. | 80 | 11 | Fibrosarcoma | 3/5 |
| | | 83 | 5·5 | Fibrosarcoma | 4/6 |
| Sodium maleate (aqueous) | 122 mg. | 104 | — | Carcinoma of thyroid* | N/A |
| | ,, | 104 | — | Carcinoma of thyroid* | N/A |
| $\beta\beta$-Dimethyltrimethylene oxide | 102 mg. | 83 | — | Fibrosarcoma | 2/6 |
| | ,, | 96 | 34 | Malignant fibrosarcoma | 1/6 |
| Penicillin G | 260 mg. | 97 | 29 | Highly malignant fibrosarcoma | 3/6 |
| | ,, | 101 | 30 | Fibrosarcoma | 0/6 |
| | ,, | 108 | 10 | Fibrosarcoma | 0/3 |
| | ,, | 78 | 16 | Fibrosarcoma | 0/6 |
| | ,, | 95 | 23 | Fibrosarcoma | 0/6 |
| | ,, | 106 | — | Interstitial cell tumour of testis* | N/A |
| Parasorbic acid | 128 mg. | 63 | 6 | Fibrosarcoma | 0/6 |
| | ,, | 66 | 15 | Fibrosarcoma with necrosis | 3/4 |
| | ,, | 71 | 37 | Fibrosarcoma with cystic degeneration | 5/6 |
| | ,, | 84 | 9 | Highly malignant sarcoma | 2/6 |
| | 12·8 mg. | 61 | 20 | Fibrosarcoma | 2/6 |
| | ,, | 76 | 34 | Malignant sarcoma | 3/6 |
| | ,, | 101 | 18 | Sarcoma | 4/5 |
| | ,, | 106 | 19 | Sarcoma | 0/6 |

* Tumours not at injection site. N/A = not attempted.

### A PRELIMINARY STUDY ON GROWTH INHIBITION BY LACTONES AND RELATED SUBSTANCES

(In collaboration with H. B. Waynforth)

In view of the selective growth inhibitory properties of certain lactones, particularly parasorbic acid (V), we have begun experiments with some of our series of carcinogenic lactones to see if they are also growth-inhibitory. These two distinct types of biological activity are known to be associated in the nitrogen mustards and epoxides, for example. Naturally occurring growth inhibiting sub-

TABLE III.—*The Growth Inhibitory Effect of Some Lactones and Related Substances on a Transplantable, Penicillic Acid Induced, Rat Sarcoma*

| Period from transplant to last dose (days) | Number of tumours | Compound administered | Daily dose (mg.) | Period of injections (days) | Mean increase in body weight (g.) | Mean tumour weight ±S.D. | Significance of difference from control (=$P$) |
|---|---|---|---|---|---|---|---|
| 31 | 6 | Control | — | 0 | +72 | 8·74±1·9 | — |
| | 6 | Penicillic acid in water | 2 | 22 | +63 | 4·16±1·2 | 0·05–0·1 |
| | 6 | Methyl protoanemonin (in water) | 5 | 22 | +63 | 6·45±0·9 | — |
| | 6 | β-Propiolactone (in water) | 2 | 22 | +61 | 9·87±2·5 | — |
| 36 | 5 | Control (oil) | — | 24 | +59 | 5·27±1·5 | — |
| | 6 | 2-Hexenoic acid in oil | 5 | 24 | +50 | 5·52±3·0 | — |
| | 6 | Dimer of 2-hexenoic acid in oil | 5 | 24 | +55 | 4·68±1·7 | — |
| | 5 | Thiodiglycollic acid in oil | 5 | 24 | +41 | 4·77±0·9 | — |
| 43 | 6 | Control (oil) | — | | +56 | 10·60±2·0 | |
| | 6 | Penicillic acid in oil | 10 | 33 (toxic) | +26 | 5·2±0·5 | 0·05 |
| | 6 | Thiodiglycollic acid in oil | 10 | 33 | +41 | 7·3±1·8 | — |
| | 4 | β-Propiolactone in oil | 10 | 33 (toxic) | +22 | 10·2±2·7 | — |

stances, presumed to be of the type of parasorbic acid (V), have been obtained from a wide range of biological sources (Heaton, 1929; Medawar, 1937). Medawar, Robinson and Robinson (1943) and Kuhn *et al.* (1943) have shown that the synthetic optically inactive lactone of structure V is capable of differential inhibition of the growth of mesenchymal (but not of epithelial) tissues *in vitro* (cf. Hauschka, 1944).

A transplantable sarcoma, which had been induced by injections of penicillic acid in the rat, was used in our studies. Groups of 6 animals were given subcutaneous transplants of this tumour and daily injections of 2 mg. to 10 mg. of selected compounds in aqueous or oily solution were begun about 10 days later. When the transplants in untreated or oil-injected rats had attained a satisfactory size the rats were killed and the tumours weighed. Care was taken to ensure that the injections were not placed in tumour tissue but in subcutaneous sites elsewhere in the rats. Animal weights were recorded daily as an indication of toxic effects resulting from the treatment, and when these were severe the daily administrations were withheld. The results are given in Table III.

Penicillic acid in two experiments inhibited tumour growth by about 50 per cent and the difference from the control tumour size was statistically significant in each case, though the degree of inhibition did not appear to be related to the dose used. There was also a depressing effect on normal body growth but the effect on growth of the tumour does not seem to be directly dependent on this, since with $\beta$-propiolactone (10 mg. daily) there was marked suppression of body growth, but not of the tumour growth. 2-Hexenoic acid lactone, and its dimeric form, as well as methylprotoanemonin also had no effect on tumour growth. Thiodiglycollic acid is not a member of the lactone series, but has been reported (Sahasrabudhe *et al.*, 1961) to inhibit tumour growth, and was included for this reason. No significant inhibition of the growth of this sarcoma was obtained with daily injections of 5 mg. and 10 mg. of thiodiglycollic acid in our experiments (Table III).

SUMMARY

1. Further lactones and chemically related compounds have been tested for carcinogenicity by means of twice weekly injections subcutaneously into rats; thus extending the earlier work of Dickens and Jones (1961).

2. A further four-membered ring compound which was carcinogenic was $\beta\beta$-dimethyltrimethylene oxide. The carcinogenicity of penicillin G, which contains a 4-membered lactam ring, was confirmed; 2 mg. doses of the sodium salt gave local tumours in 5 of 11 further rats so treated. $\beta$-Hydroxypropionic acid, formed by hydrolysis of $\beta$-propiolactone was not carcinogenic.

3. Among 5-membered ring systems, penicillic acid was re-tested in 0·1 mg. doses in oil and 2 mg. in aqueous solution. Both gave local tumours, the latter in 4 of 5 surviving rats. Maleic anhydride, but not sodium maleate, also produced local tumours at the injection site.

4. The optically active 6-membered $\alpha\beta$-unsaturated hexenolactone, (+)-parasorbic acid, isolated from mountain ash berries, was carcinogenic both at the 2 mg. (local tumours in 4 of 5 rats injected) and 0·2 mg. (4 tumours in 6 rats) dose. This substance is of special interest in view of its wide distribution in nature and its selective growth inhibitory properties.

5. The above local tumours were all at or near the injection site, were histologically malignant sarcomas, and many of them were successfully transplanted into other rats. Repeated injection of the arachis oil, which was used as solvent in most experiments, failed to yield any local tumours.

6. Tests for possible tumour-growth inhibiting ability were made using a transplanted rat sarcoma originally produced by penicillic acid. None of the following caused significant inhibition : $\beta$-propiolactone, methyl protoanemonin, 2-hexenoic acid-$\gamma$-lactone, the dimer of this lactone, or thiodiglycollic acid. On the other hand, penicillic acid (2 mg. doses in water or 10 mg. doses in oil) caused 50 per cent inhibition of tumour growth in two separate experiments.

We wish to thank Dr. A. C. Thackray for his kindness in giving opinions on the histological findings. Gifts of materials from Professor J. H. Birkinshaw, Professor G. R. Clemo, Professor A. Steinhofer and Dr. A. L. Walpole are gratefully acknowledged. Technical assistance was provided by Mr. S. Graves and Miss Judith Cooke and Mr. P. A. Runnicles kindly prepared the photographs.

This work was supported by a block grant made to the Medical School by the British Empire Cancer Campaign.

### REFERENCES

DICKENS, F.—(1962) 'On Cancer and Hormones', University of Chicago Press, pp. 107–120.

*Idem* AND JONES, H. E. H.—(1961) *Brit. J. Cancer*, **15**, 85.

DOEBNER, O.—(1894) *Ber. Dtsch. chem. Ges.*, **27**, 345.

HAUSCHKA, T. S.—(1944) *Nature, Lond.*, **154**, 769.

HEATON, T. B.—(1929) *J. Path. Bact.*, **32**, 565.

HOFMANN, A. W.—(1859) *Liebigs Ann.*, **110**, 129.

KUHN, R. AND JERCHEL, D.—(1943) *Ber. Dtsch. chem. Ges.*, **76**, 410.

*Idem*, MOEWUS, F., MÖLLER, E. F. AND LETTRÉ, H.—(1943) *Naturwissenschaften*, **31**, 468.

MEDAWAR, P. B.—(1937) *Quart. J. exp. Physiol.*, **27**, 147.

*Idem*, ROBINSON, G. M. AND ROBINSON, R.—(1943) *Nature Lond.*, **151**, 195.

MORGAN, E. J. AND FRIEDMANN, E.—(1938) *Biochem. J.*, **32**, 733.

ROE, F. J. C. AND GLENDENNING, O. M.—(1956) *Brit. J. Cancer*, **10**, 357.

SAHASRABUDHE, M. B., NARURKA, M. V., KOTNIS, L. B., TILAK, B. D., SHAH, L. G. AND GOGTE, V. N.—(1961) *Nature, Lond.*, **191**, 388.

WALPOLE, A. L., ROBERTS, D. C., ROSE, F. L., HENDRY, J. A. AND HORNER, R. F.—(1954) *Brit. J. Pharmacol.*, **9**, 306.